# Independent Prognostic Value of Intratumoral Heterogeneity and Immune Response Features by Automated Digital Immunohistochemistry Analysis in Early Hormone Receptor-Positive Breast Carcinoma

**DOI:** 10.3389/fonc.2020.00950

**Published:** 2020-06-16

**Authors:** Dovile Zilenaite, Allan Rasmusson, Renaldas Augulis, Justinas Besusparis, Aida Laurinaviciene, Benoit Plancoulaine, Valerijus Ostapenko, Arvydas Laurinavicius

**Affiliations:** ^1^Department of Pathology, Forensic Medicine and Pharmacology, Faculty of Medicine, Institute of Biomedical Sciences, Vilnius University, Vilnius, Lithuania; ^2^National Centre of Pathology, Affiliate of Vilnius University Hospital Santaros Clinics, Vilnius, Lithuania; ^3^ANTICIPE, Inserm (UMR 1086), Cancer Center F. Baclesse, Normandy University, Caen, France; ^4^Department of Breast Surgery and Oncology, National Cancer Institute, Vilnius, Lithuania

**Keywords:** immunohistochemistry, digital pathology, breast cancer, intratumoral heterogeneity, progesterone receptor, Ki67, SATB1, immune response

## Abstract

Immunohistochemistry (IHC) for ER, PR, HER2, and Ki67 is used to predict outcome and therapy response in breast cancer patients. The current IHC assessment, visual or digital, is based mostly on global biomarker expression levels in the tissue sample. In our study, we explored the prognostic value of digital image analysis of conventional breast cancer IHC biomarkers supplemented with their intratumoral heterogeneity and tissue immune response indicators. Surgically excised tumor samples from 101 female patients with hormone receptor-positive breast cancer (HRBC) were stained for ER, PR, HER2, Ki67, SATB1, CD8, and scanned at 20x. Digital image analysis was performed using the HALO™ platform. Subsequently, hexagonal tiling was used to compute intratumoral heterogeneity indicators for ER, PR and Ki67 expression. Multiple Cox regression analysis revealed three independent predictors of the patient's overall survival: Haralick's texture entropy of PR (*HR* = 0.19, *p* = 0.0005), Ki67 Ashman's D bimodality (*HR* = 3.0, *p* = 0.01), and CD8+SATB1+ cell density in tumor tissue (*HR* = 0.32, *p* = 0.02). Remarkably, the PR and Ki67 intratumoral heterogeneity indicators were prognostically more informative than the rates of their expression. In particular, a distinct non-linear relationship between the rate of PR expression and its intratumoral heterogeneity was observed and revealed a non-linear prognostic effect of PR expression. The independent prognostic significance of CD8+SATB1+ cells infiltrating the tumor could indicate their role in anti-tumor immunity. In conclusion, we suggest that prognostic modeling, based entirely on the computational image-based IHC biomarkers, is possible in HRBC patients. The intratumoral heterogeneity and immune response indicators outperformed both conventional breast cancer IHC and clinicopathological variables while markedly increasing the power of the model.

## Introduction

Gene expression profiling studies have identified several subtypes of breast cancer (BC) distinguished by the expression of hormone receptor, cell proliferation, and human epidermal growth factor receptor 2 (HER2) genes (1–3). The subtypes have been associated with different biological behavior and different responses to treatment (4–6). The current clinical practice is mainly based on immunohistochemistry (IHC) for estrogen receptors (ER), progesterone receptors (PR), HER2, and Ki67. In fact, the American Society of Clinical Oncology (ASCO) and the College of American Pathologists (CAP) guidelines, updated in 2020, recommended to continue the use of IHC of these biomarkers as the primary method to categorize BC cases, predict disease outcomes and guide treatment decisions (7, 8).

Combined IHC biomarkers have been proposed for better prognostic modeling of BC. In 2005, Abd El-Rehim et al. (9) demonstrated that combined multiple protein expression profiles from visual IHC assessment of 25 relevant biomarkers in tissue microarrays (TMA) might be used as an alternative to gene expression profiling methods. Subsequently, Soria et al. (10) applied hierarchical clustering to reduce this number to only 10 biomarkers. In 2011, Cuzick et al. (11) proposed to combine semi-quantitative scoring of ER, PR, Ki67 and HER2 IHC into an “IHC4 score”; further studies (11, 12) confirmed that the IHC4 score along with clinicopathological features provided similar prognostic information compared to gene expression profiling tests such as the Oncotype DX (Genomic Health Inc., Redwood City, CA) or Prosigna (NanoString Technologies, Seattle, WA). However, the IHC analyses were criticized for a lack of reliability and poor reproducibility (13–19).

Advances in digital image analysis (DIA) and computational pathology have opened new opportunities for more accurate and reproducible measurements (20–26). In 2002, Camp et al. (27) developed a set of algorithms for automated quantification of protein expression with pixel-based tissue segmentation using fluorescent labels for cytokeratin and α-catenin. They found that automated analysis of ER has better reproducibility and higher significance of prognostic information than conventional pathologist-based scoring (27). Jakobsen et al. (28) compared visual HER2 IHC scoring with quantitative measurements by DIA and detected that automated analysis could significantly (67%) reduce the proportion of HER2 equivocal cases without affecting false-negative rate. Likewise, Stalhammar et al. (29) demonstrated that tumor subtyping by a combination of ER, PR, Ki67, and HER2 manual scores was prognostically and predictively inferior when compared to DIA of corresponding biomarkers and concluded that DIA is a robust, reproducible and less time-consuming alternative to the manual scoring in BC.

Besides the benefits of better accuracy and precision, indicators based on large scale data extraction by DIA enable application of more powerful statistical processing methods when combining the informative value of the various IHC markers (30). In 2015, Laurinavicius et al. (31) demonstrated independent prognostic power of special AT-rich sequence-binding protein 1 (SATB1) and Ki67 ratio in a study of 10 IHC markers in BC TMA obtained by automated DIA and multivariate statistical modeling of the data. In 2019, Abubakar et al. (32) reported that the IHC4 method retained its prognostic value when based on DIA quantification measurements; furthermore, a combined quantitative measure of biomarkers outperformed the conventional dichotomous IHC scoring in two independent BC patient cohorts (32).

DIA applications in IHC have also enabled new ways to measure intratumoral heterogeneity of the biomarker expression. In particular, the issue of Ki67 hotspot detection and assessment has been addressed by many investigators (15, 16, 22, 23, 33–35). Stalhammer et al. (29) showed that the error rate of a pathologist's stratification into distinct BC subtypes based on visual assessment of Ki67 expression could be reduced by 12% using DIA with automated hotspot detection. However, a rigorous definition of hotspots has not been established: hotspots may vary in size, shape, number and difficult to ascertain in homogeneous cases (23, 36, 37). Conversely, intratumoral heterogeneity assessment, based on systematic hexagonal grid subsampling of the DIA data, is a clear statistical definition of heterogeneity and (34) revealed that Ki67 intratumoral heterogeneity was more prognostically important than its rate of expression *per se*.

Recent advances in cancer immunotherapy have opened new challenges in the search for reliable prognostic and predictive biomarkers of the antitumor immune response in the context of tumor microenvironment (TME) (38–42). Chronic inflammation properties, one of the hallmarks of cancer, have been shown to contribute to tumor initiation, progression, and metastasis (43–46). Tumor-infiltrating lymphocytes (TIL) have been associated with a better prognosis and clinical outcome (47–49). An abundance of CD3+, CD4+, CD8+, and CD45RO+ TIL has been reported to be a feature of efficient immune response in several cancers (48, 50–54). While a higher density of cytotoxic CD8+ T cells has been linked to a positive anti-tumor effect (55–59), the CD8+PD-1+ subset of TIL is associated with worse prognosis and therapy outcomes (60–63). This could be related to the interaction between programmed death 1 (PD-1) receptor and programmed death-ligand 1 (PD-L1) which leads to the impairment and exhaustion of TIL and inhibition of immune responses against tumor cells (60, 64). This has made the blockade of PD-1 or PD-L1 an important therapeutic approach in treating tumors (65–70). However, a rather low proportion of patients benefit from this immunotherapy (65–71). A comprehensive TIL study in BC has demonstrated that CD8+ T cells can retain cytolytic activity despite PD-1 expression and highlighted the need for a better understanding of how PD-1 pathway could induce T cell exhaustion (72). Recently, a link between PD-1, SATB1 and cancer immunity has been reported (73, 74) suggesting that SATB1 may be a novel biomarker for prediction of the functional properties of T cells in the TME.

In this study of hormone receptor-positive BC (HRBC), we explored the prognostic value of IHC, performed on full-face surgical excision slides, by combining DIA data from multiple IHC biomarkers, along with their intratumoral heterogeneity indicators and tissue immune response properties, in the context of conventional clinicopathologic variables. We found that the DIA IHC data alone generated the most significant prognostic model of the patient overall survival (OS), represented by three independent features: PR entropy, Ki67 bimodality, and CD8+SATB1+ cell density in the tumor tissue. Remarkably, the intratumoral heterogeneity indicators of PR and Ki67 were prognostically more informative than the rates of their expression. The independent prognostic role of CD8+SATB1+ TIL is suggestive of the potential utility of this biomarker in the context of cancer immunotherapy.

## Materials and Methods

### Study Population and Tumor Characteristics

Surgically excised tumor samples from 101 patients with HRBC were used. The same patient cohort was investigated in the previous TMA studies (30, 31), with extended follow-up period now. Briefly, the patients were treated at the National Cancer Institute (Vilnius, Lithuania) and tested at the National Center of Pathology (Vilnius, Lithuania) from 2007 to 2009. The OS follow-up period ranges from 17 to 143 months, with a median of 135 months. The demographic, clinicopathological and follow-up characteristics of the patient cohort are summarized in Table 1. The study was approved by the Lithuanian Bioethics Committee (reference number: 40, date 2007-04-26, updated 2017-09-12). Informed written consent was obtained from all patients before study entry.

**Table 1:** Patient and tumor clinicopathologic parameters.

**Clinicopathologic parameters**	
Patients	101 (100%)
**Age, years**	
Median	59
Range	27–87
**Sex**	
Female	101 (100%)
Male	0
**Follow up, months**	
Median	135
Range	17–143
Deceased	24 (23.8%)
**Histological grade (G)**	
G1	23 (22.8%)
G2	47 (46.5%)
G3	31 (30.7%)
**Tumor invasion stage (pT)**	
T1	55 (54.5%)
T2	46 (45.5%)
T3	0
T4	0
**Lymph node metastasis status (pN)**	
N0	54 (53.5%)
N1	35 (34.6%)
N2	9 (8.9%)
N3	3 (3.0%)
**Treatment**	
Hormone therapy	88 (87.1%)
Chemotherapy	61 (60.4%)
Radiotherapy	85 (84.2%)
Trastuzumab therapy	7 (6.9%)

### Immunohistochemistry

Formalin-fixed paraffin-embedded full-face sections of surgically excised tumors were cut 3 μm thick and mounted on positively charged slides (six sections per case). A Roche Ventana BenchMark ULTRA automated staining system (Ventana Medical Systems, Tucson, Arizona, USA) was used to perform the IHC staining. ER, PR, HER2, Ki67, HIF1α, and SATB1 were detected using the ultraView Universal DAB Detection kit, and CD8 was visualized using the ultraView Universal Alkaline Phosphatase Red Detection kit (Ventana Medical Systems, Tucson, Arizona, USA). IHC was performed using ready-to-use antibodies for ER, PR, HER2 (SP1, 1E2, 4B5, respectively, Ventana (Tucson, Arizona, USA), Ki67 (MIB-1, DAKO (Glostrup, Denmark), dilution 1:200), HIF1α (EP118, Epitomics (San Mateo, USA), dilution 1:200) and double IHC for SATB1 (SP287, Abcam (Cambridge, United Kingdom), dilution 1:250) and CD8 (C8/144B, DAKO, dilution 1:1100). The sections were counterstained with Mayer's hematoxylin.

### Digital Image Acquisition and Analysis

**Figure 1:**
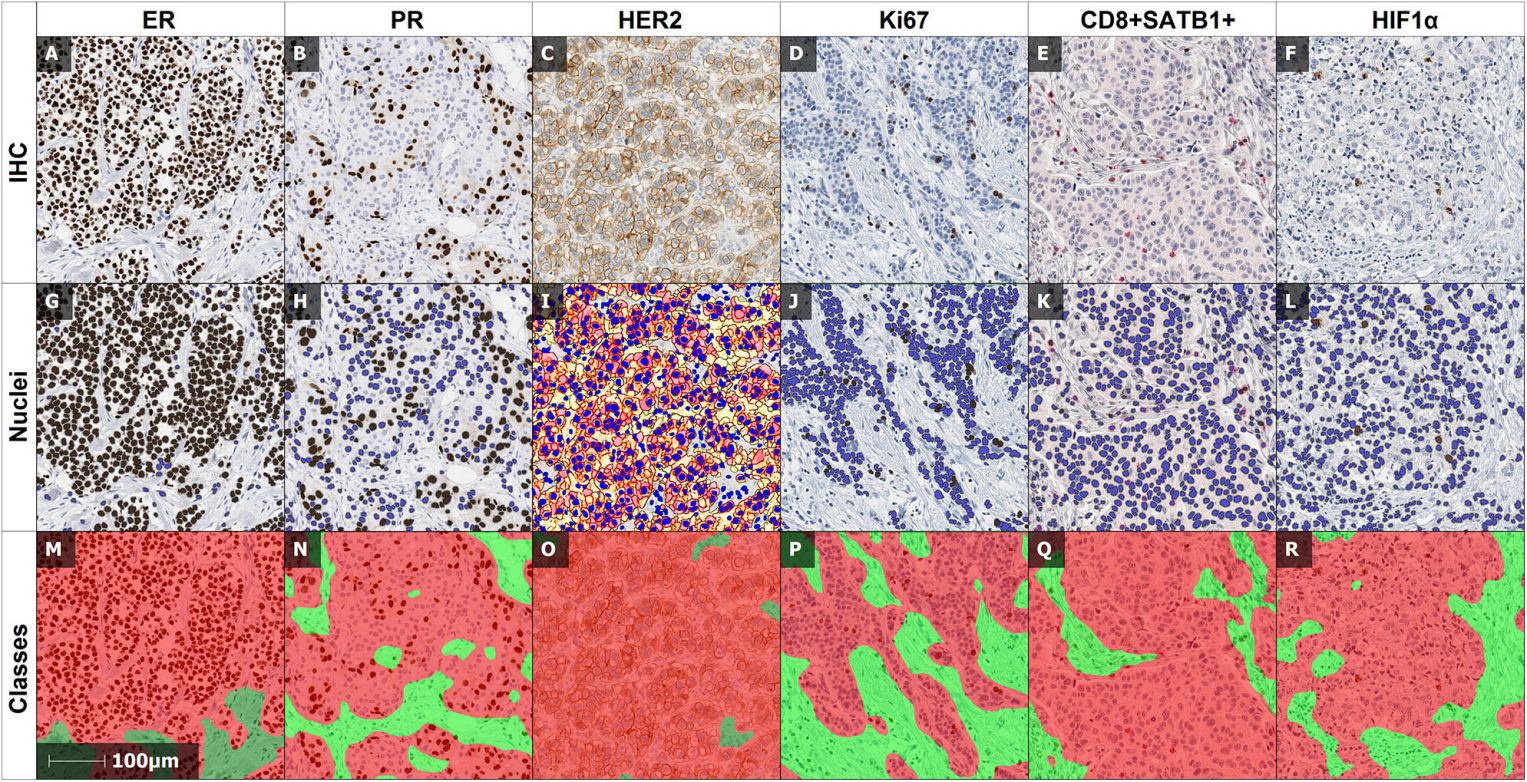
Examples of IHC and DIA output images. **(A,G,M)**: IHC and corresponding DIA outputs of ER, **(B,H,N)** of PR, **(C,I,O)** of HER2, **(D,J,P)** of Ki67, **(E,K,Q)** of double IHC of CD8 and SATB1, and **(F,L,R)** of HIF1α. The nuclear algorithms mark positive (brown), and negative (blue) cells: **(G)** of ER, **(H)** of PR, **(J)** of Ki67 and cytoplasmic/nuclear algorithm marks positive HIF1α cells **(L)**, HER2 algorithm marks the positive cells of HER2 **(I)** with color masks according to staining intensity (negative—blue, week positive—yellow, moderate positive—orange, intense positive—red). Multiplex algorithm of double IHC **(E)** separates positive SATB1 (brown), positive CD8 (red) and negative (blue) cells. **(M–R)**: illustrate the automated detection of the tumor (red) and stroma (green) compartments by the HALO AI tissue classifier.

The IHC slides were scanned with a ScanScope XT Slide Scanner (Leica Aperio Technologies, Vista, CA, USA) at 20× objective magnification (0.5 μm pixel resolution). DIA was performed with the HALO software (version 3.0311.174; Indica Labs, Corrales, New Mexico, United States): the HALO AI tissue classifier was trained to segment tumor tissue, stroma, and background (consisting of necrosis, artifacts, and glass); HALO Multiplex IHC algorithm (version 1.2) was used to detect and extract coordinates of nuclear ER, PR, Ki67, SATB1, and cytoplasmic CD8 and HIF1α positive cells, while HALO HER2 algorithm (version 1.1) was used for HER2 positive cells. Examples of IHC and DIA analysis output images are presented in Figure 1.

### Computation of IHC Indicators

The set of IHC indicators used in the prognostic models included: (1) per-case global quantities (percentages of ER, PR, Ki67 positive cells and HER2 2+ and 3+ in the tumor compartment), (2) intratumoral heterogeneity of ER, PR, and Ki67 positivity, (3) immune response properties represented by the densities of CD8+ and CD8+SATB1+ cells in tumor and stroma compartments, and (4) hypoxia-inducible properties represented by the percentage of HIF1α positive cells in tumor and stroma compartments.

Indicator sets 1, 3 and 4 were readily extracted from the HALO DIA data for each digitized slide. The intratumoral heterogeneity indicators were computed using the hexagonal tiling methodology as previously reported (30, 31, 35). Briefly, the HALO DIA data were subsampled by a randomly positioned hexagonal grid (hexagon side length 257 μm). Based on the cell coordinates obtained by the DIA, the number of positive and negative cells of all biomarkers were counted inside each hexagon. Hexagons containing fewer than 50 cells were regarded as insufficient sampling and discarded from further analyses. Since low expression and low dynamic range was observed for HER2, CD8+, CD8+SATB1+, and HIF1α, no heterogeneity indicators were extracted for these biomarkers and were instead quantified in the stroma and/or tumor compartment. The percentages of ER, PR, and Ki67 were calculated for each hexagon, and subsequently ranked linearly into ten intervals (0–10%, >10–20%, etc.) for computation of a co-occurrence matrix. Heterogeneity markers were extracted from the co-occurrence matrix as Haralick's texture indicators (contrast, dissimilarity, entropy, energy, homogeneity). The bimodality indicator Ashman's D (AshD) was computed for the intratumoral distributions of ER, PR and Ki67 expression in the hexagonal grids as described in detail previously (31).

### Statistical Methods

Summary statistics and distribution analyses were performed with significance tests based on one-way ANOVA. Bonferroni's *post hoc* test and Welch's *t-*tests were used for pairwise and homogeneity of variances comparisons, respectively. Fisher's exact test was used to determine the associations between categorical variables. Highly correlated (*r* > 0.90) indicators were eliminated to avoid multicollinearity or singularity in multivariate survival analysis. Due to a limited cohort size overfitting was minimized by leave-one-out cross-validation (75), the most frequent variable subsets were further tested in the survival prediction models. Subsequently, a factor analysis was performed for seven IHC biomarkers with factors retained based on an eigenvalue > 1; orthogonal varimax rotation of the initial factors was used. A cut-off value for each indicator was determined by Cutoff Finder software (Charité University, Berlin, Germany) (76) to test univariate OS predictions. The OS distributions were estimated using the Kaplan–Meier method followed by log-rank testing to assess the statistical significance of differences between the stratified groups. Cox proportional hazards analysis was performed to test independent prognostic significance of the IHC indicators in the context of clinicopathologic variables. Statistical analyses were performed using SAS (version 9.4; SAS Institute Inc., Cary, North Carolina, USA). The statistical significance level was set at *p* < 0.05. Plots were produced using R (version 3.4.4).

## Results

### Summary Statistics

Summary statistics of the IHC indicators are presented in Supplementary Table 1. Of note, one-way ANOVA and Bonferroni's *post hoc* test of immune response and hypoxia-inducible indicators showed that the percentage of HIF1α and density of CD8+ and CD8+SATB1+ were significantly higher in stroma than in the tumor compartment (*p* < 0.0001) (data not shown).

### Factor Analysis of IHC Indicators

**Figure 2:**
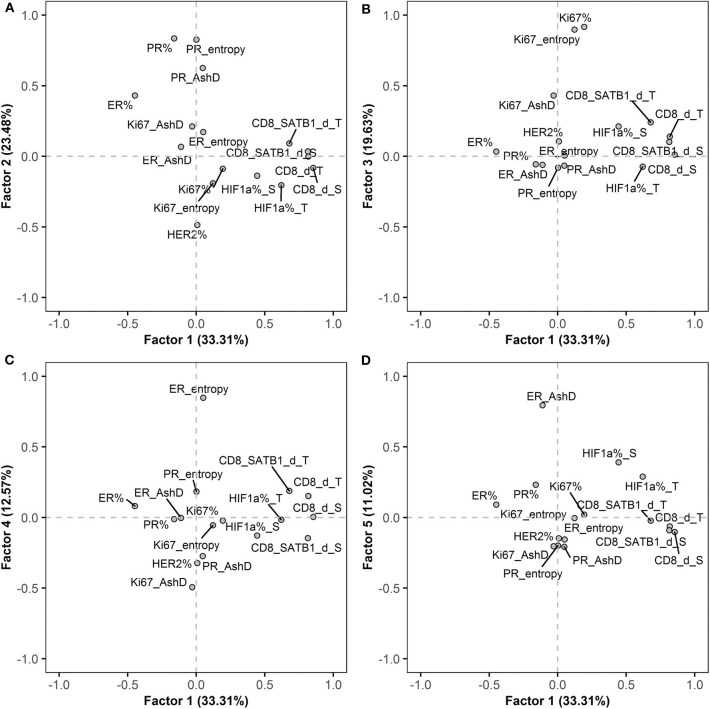
Rotated factor pattern of the IHC indicators: AshD, Ashman's D; d, density; S, stroma compartment; T, tumor compartment. **(A)** The loading of factors 1 and 2; **(B)** factors 1 and 3; **(C)** factors 1 and 4 and **(D)** factors 1 and 5 are plotted (*n* = 101).

To explore inherent correlations between the IHC indicators, a factor analysis was performed for a set of conventional BC, immune response, hypoxia-inducible and intratumoral heterogeneity (ER, PR and Ki67 AshD bimodality and Haralick's texture entropy) indicators. Five orthogonally independent factors were extracted, the rotated factor loadings are presented in Supplementary Table 2 and the pattern of five factors is plotted in Figure 2. Factor 1 was characterized by strong loadings of CD8+ and CD8+SATB1+ cell densities within the tumor and stroma compartments, factor 2 by the percentage of PR, PR AshD and entropy indicators; factor 3 by the percentage of Ki67 and Ki67 entropy indicators; factor 4 by ER entropy and factor 5 by ER bimodality. Altogether, the five factors explained 64% of the variance in the dataset and indicated orthogonally independent latent factors governing the variation of IHC indicators.

### Prognostic Value of IHC and Clinicopathologic Indicators in Univariate Analyses

Kaplan-Meier survival analyses with hazard ratio (HR) and log-rank test were performed to estimate the prognostic potential of the IHC and clinicopathologic indicators. The main results are summarized in Table 2; the results in their entirety are presented in Supplementary Figure 1. Higher PR and HER2 expression in the tumor tissue, CD8+ and CD8+SATB1+ cell densities in the stroma and tumor tissue, ER and PR entropy, contrast, dissimilarity and PR AshD are associated with higher OS probabilities. Worse OS is associated with higher ER, ER and PR energy, homogeneity and Ki67 AshD. No significant stratifications were obtained for the Ki67 and HIF1α expression, histological grade, T stage, lymph node status, ER AshD, Ki67 energy, homogeneity, entropy, contrast and dissimilarity. Higher patient age at the time of surgery was associated with worse OS (*HR* = 2.45, *p* = 0.039).

**Table 2:** Kaplan-Meier estimates using log-rank test for overall survival in relation to IHC, intratumoral heterogeneity and clinicopathologic indicators.

**Univariate regression analysis**				**Intratumoral heterogeneity indicators**			
	**HR**	**95% CI**	***p*-value**		**HR**	**95% CI**	***p*-value**
**Conventional breast cancer indicators**							
ER%	3.11	1.16–8.34	0.017	ER_energy	4.56	1.36–15.31	0.007
PR%	0.30	0.14–0.68	0.002	ER_homogeneity	3.40	1.52–7.62	0.002
Ki67%	2.13	0.80–5.71	0.120	ER_entropy	0.09	0.01–0.68	0.003
HER2%	0.39	0.17–0.92	0.025	ER_contrast	0.31	0.14–0.71	0.004
Immune response indicators				ER_dissimilarity	0.32	0.14–0.70	0.003
CD8_d_S	0.31	0.11–0.82	0.013	ER_AshD	2.11	0.72–6.17	0.160
CD8_d_T	0.23	0.10–0.57	0.0005	PR_energy	5.36	2.12–13.52	<0.0001
CD8_SATB1_d_S	0.32	0.13–0.81	0.011	PR_homogeneity	4.88	2.02–11.79	0.0001
CD8_SATB1_d_T	0.26	0.11–0.57	0.0004	PR_entropy	0.21	0.08–0.52	0.0002
Hypoxia-inducible indicators				PR_contrast	0.22	0.09–0.56	0.0005
HIF1α%_S	0.43	0.15–1.26	0.11	PR_dissimilarity	0.15	0.05–0.44	<0.0001
HIF1α%_T	0.46	0.16–1.35	0.15	PR_AshD	0.32	0.14–0.71	0.003
Clinicopathological variables				Ki67_energy	0.48	0.20–1.17	0.100
G stage (G1–2 vs. G3)	1.20	0.52–2.81	0.670	Ki67_homogeneity	0.46	0.19–1.12	0.079
T stage (T1 vs. T2)	0.99	0.45–2.22	0.986	Ki67_entropy	2.06	0.85–4.98	0.100
N status (N0 vs. N1–3)	2.17	0.95–4.97	0.07	Ki67_contrast	2.11	0.93–4.74	0.066
Age (≤ 59 vs. > 59)	2.45	1.05–5.73	0.039	Ki67_dissimilarity	2.16	0.89–5.21	0.079
–	–	–	–	Ki67_AshD	2.48	1.09–5.68	0.026

*AshD, Ashman's D; d, density; S, stroma compartment; T, tumor compartment; HR, hazard ratio; CI, confidence interval*.

### Independent Predictors of OS

**Figure 3:**
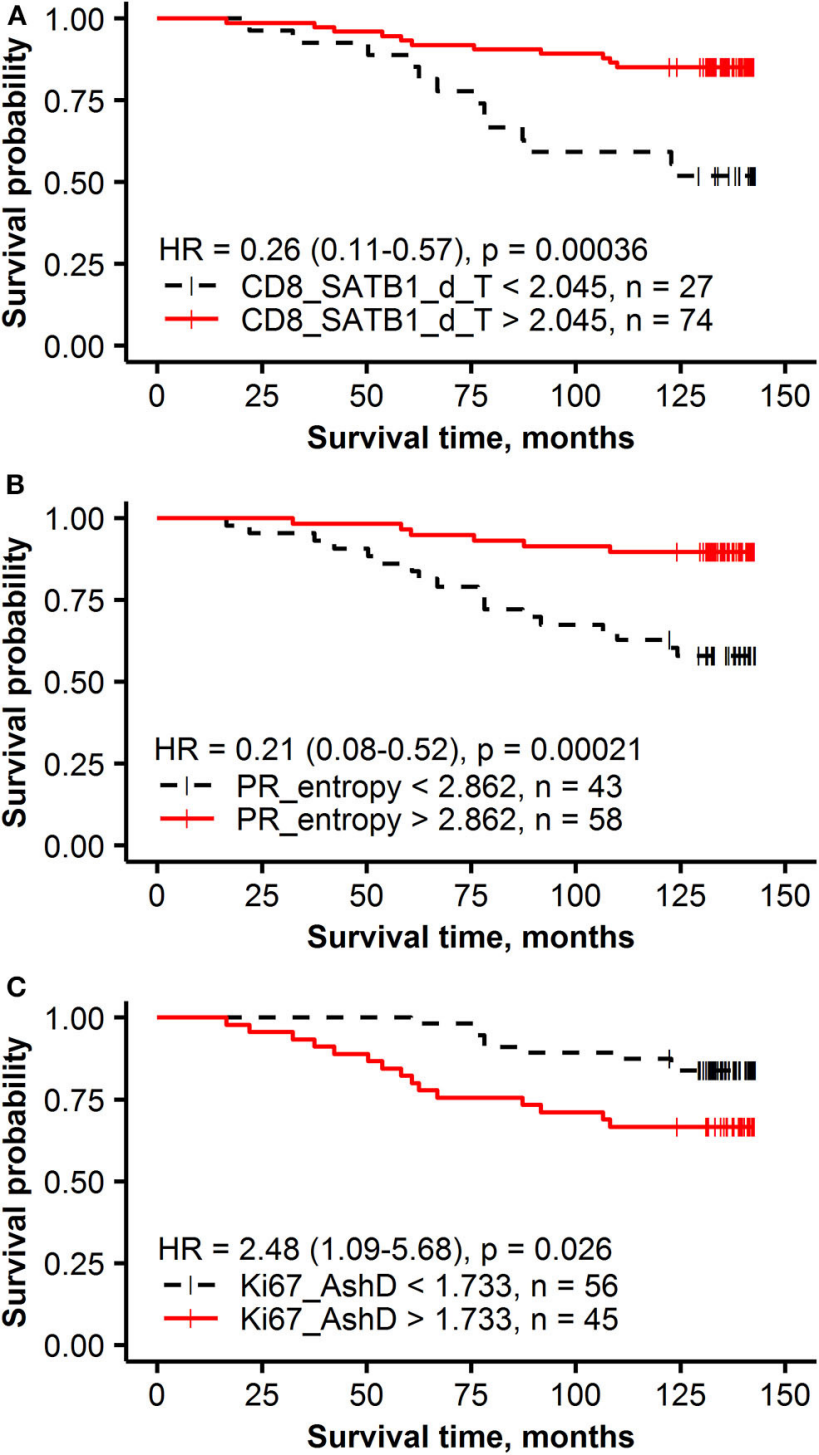
Kaplan-Meier survival plots with hazard ratio and log-rank test for correlation of IHC and intratumoral heterogeneity indicators with overall survival: **(A)** the density of CD8+SATB1+ in the tumor compartment (T), **(B)** PR entropy, **(C)** Ki67 Ashman's D (AshD).

The independent prognostic value of the global IHC biomarker expression rates, their intratumoral heterogeneity, and immune response indicators was tested by multivariate Cox regression analysis, including conventional clinicopathologic characteristics (Table 3). To estimate the added prognostic value of the novel IHC indicators, the analyses were performed in 2 datasets: Model 1 was generated from a subset consisting of the age group, pathology characteristics (pT, pN status and histological grade) and the global IHC DIA indicators (ER, PR, HER2 and Ki67 expression rates in the tumor compartment). Model 2 was obtained by supplementing the data set with the intratumoral heterogeneity and immune response indicators (Table 3). Model 1 revealed two independent factors of worse OS—lower PR expression and lymph node involvement. Model 2 showed a remarkable increase of the statistical power likelihood ratio (LR), 27.67 compared to 12.23 of Model 1 based exclusively on three novel IHC indicators: better OS was predicted by higher CD8+SATB1+ cell density in the tumor compartment and higher entropy (intratumoral heterogeneity) of PR expression; worse OS was predicted by the bimodality (AshD) of Ki67 expression in the tumor tissue. Prognostic stratifications for these indicators are presented in Figure 3.

**Table 3:** Statistics of multivariate Cox regression analyses for correlation of IHC, intratumoral heterogeneity and immune response indicators with overall survival.

**Multivariate regression analysis**			
	**HR**	**95% CI**	***p*****-value**
**Model 1 (LR: 12.23**, ***p*** **=** **0.0022)**			
N status (N0 vs. N1–3)	2.30	1.01–5.28	0.0485
PR%	0.29	0.13–0.66	0.0028
**Model 2 (LR: 27.67**, ***p<*** **0.0001)**			
CD8_SATB1_d_T	0.30	0.13–0.67	0.0035
PR_entropy	0.22	0.08–0.56	0.0015
Ki67_AshD	3.26	1.40–7.61	0.0062

*AshD, Ashman's D; d, density; T, tumor compartment; HR, hazard ratio; CI, confidence interval; LR, likelihood ratio*.

### Nonlinear Relationship Between PR Expression and Its Intratumoral Heterogeneity

**Figure 4:**
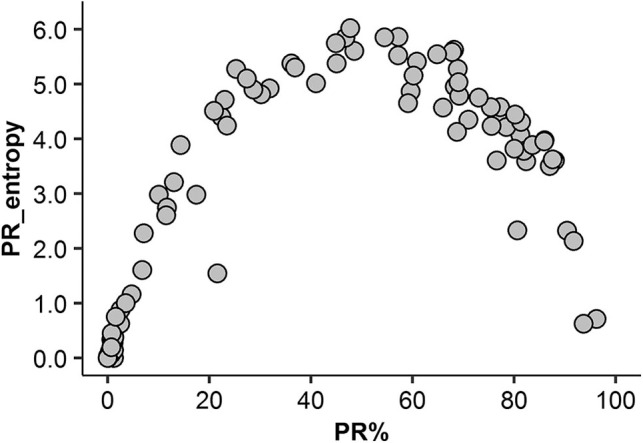
Non-linear association between the rate of PR expression and its intratumoral heterogeneity (entropy).

A non-linear relationship between the rate of PR expression and its intratumoral heterogeneity (entropy) was detected: high PR entropy was observed within the PR expression range from 20 to 80% (Figure 4). Importantly, neither of these two variables were significantly associated with other patient or tumor characteristics (data not shown). Of note, only a weak linear correlation between ER entropy and PR entropy was found (*r* = 0.31, *p* = 0.0017).

### Prognostic Value of PR Expression Rates Revealed by Its Heterogeneity Property

**Figure 5:**
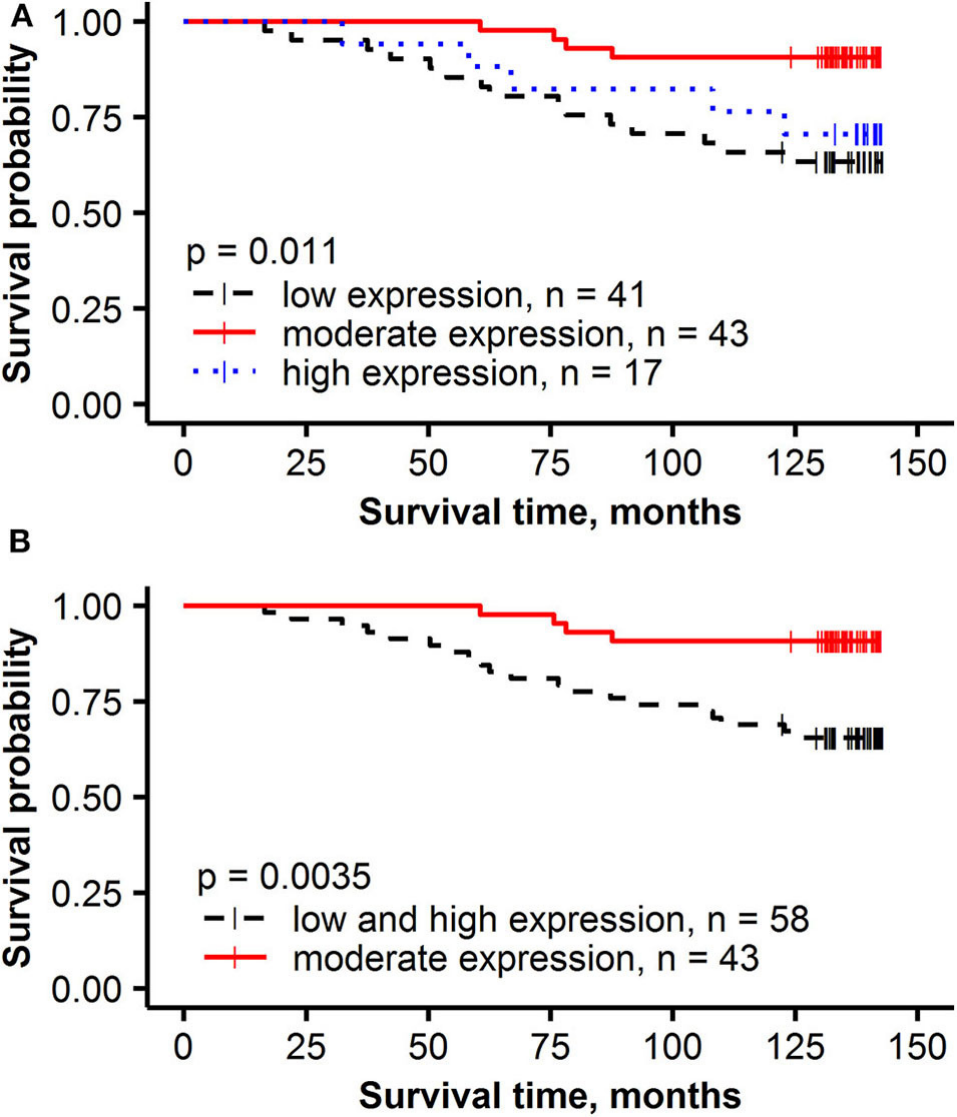
Kaplan-Meier survival plots with hazard ratio and log-rank test for correlation of PR% groups with overall survival: **(A)** low expression (< 20%), moderate expression (20–80%) and high expression (higher than 80%), **(B)** low and high expression (< 20% or higher than 80%) and moderate expression (20–80%).

To investigate the impact the non-linear relationship between PR expression rate and its intratumoral heterogeneity has on the prognostic stratification, the patients were stratified into three groups: low expression (<20%) low entropy, moderate expression (20–80%) high entropy and high expression (higher than 80%) low entropy. Tumors with moderate expression of PR (20–80%) were associated with the best OS (91% OS probability after 143 months), followed by high (>80%) expression (71% OS) and low (<20%) expression (63% OS) (Figures 5A,B).

## Discussion

Our study demonstrates the benefits of combined IHC image-based biomarker prognostic modeling and is important in several aspects: (1) the multidimensional IHC prognostic model was achieved solely from the IHC DIA data and reflected three biologic features of HRBC (PR expression, proliferation rate, immune response) and outperformed standard clinicopathologic parameters in the dataset tested; (2) intratumoral heterogeneity indicators of PR and Ki67 expression were prognostically more informative than the rates of their expression; (3) supplementing the IHC DIA results with intratumoral heterogeneity analytics markedly increased the power of the prognostic model. Overall, the study demonstrated for the first time independent prognostic value of intratumoral PR heterogeneity and intratumoral CD8+SATB1+ cell density in early HRBC.

The study was performed on full-face surgical excision sections and essentially confirms and extends the findings of a previous study based on TMA with automated IHC DIA in the same patient cohort with a shorter follow-up period (31). It also confirms the positive prognostic value of SATB1 expression in the tumor tissue as reported previously (31). However, by adding the CD8 marker to the study, we find more specifically that the prognostic impact of SATB1 is related to the density of intratumoral CD8+SATB1+ cells. Although the expression of SATB1 alone could be noted in some cancer cells in a few cases, it was not sufficient to obtain its prognostic value (data not shown). On the other hand, the density of CD8+ cells in tumor compartment revealed positive prognostic value (*HR* = 0.23, *p* = 0.00047); however, it was outperformed by CD8+SATB1+ in the multivariate prognostic model (*HR* = 0.30, *p* = 0.0035).

The biological and prognostic significance of SATB1 expression in malignancies remains controversial. Studies by Cai et al. (77) and Han et al. (78) revealed that SATB1 regulates the expression of more than 1,000 genes which are predominantly associated with cell adhesion, proliferation, cell cycle invasion, migration and apoptosis and confirmed that overexpression of SATB1 is associated with aggressive BC phenotype. In contrast to these studies, Iorns et al. (79) reported that SATB1 transcript levels acquired no function in BC pathogenesis, while Selinger et al. (80) demonstrated that the loss of SATB1 is associated with a worse prognosis in lung squamous cell carcinoma. Gene expression experiments have confirmed that SATB1 regulates around 300 of T cell genes (81–83) and initial studies have indicated that SATB1 might play a vital role in anti-tumor T cell responses (74, 84). Stephen et al. (74) demonstrated in mouse models that SATB1 regulates T cell exhaustion marker PD-1, T-cell proliferative capacity and effector function. They have shown that decreased expression of SATB1 leads to a 40-fold increased expression of PD-1 and impaired anti-tumor immunity. Temporal SATB1 expression changes were noted by Nüssing et al. (84) in human tissue samples (blood, thymus, spleen, lymph nodes) supporting the notion that downregulation of SATB1 may lead to T cell dysfunction. In our study, besides the independent prognostic value of tumoral CD8+SATB1+ cell infiltrates, we found that SATB1 was more frequently expressed in CD8+ cells in stroma than in tumor tissue (26 and 21%, *p* < 0.0001, respectively). We suggest, therefore, that our findings further support the hypothesis that SATB1 expression can be a feature of activated CD8+ cells and may serve as a potential immune response biomarker in malignancies.

The role of TIL has been investigated in BC and association with better prognosis was demonstrated in ER-negative, HER2-positive and triple-negative breast tumors (85–88). However, contradicting findings on the clinical relevance of TIL have been reported in ER-positive and HER2-negative cancer (85, 89–91). A large study (89) of 12,439 BC patients, found no association between survival and manually quantified CD8+ T cells while Sobral-Leite et al. (92), who analyzed TIL based on IHC and DIA, detected that CD8+ T cells were associated with worse clinical outcome and *PIK3CA* mutations in ER-positive BC. Lee at al. (93) also indicated that TIL might have a different prognostic impact across BC subtypes, although their study results were not statistically significant. Our study, based on DIA, retrieved the prognostic value of both stromal and tumoral CD8+ lymphocytes (*HR* = 0.31, *p* = 0.013 and *HR* = 0.23, *p* = 0.00047, respectively) in univariate analyses and tumoral CD8+ lymphocytes (*HR* = 0.39, *p* = 0.04) in multiple Cox regression model (not shown); however, tumoral CD8+SATB1+ cells further increased the prognostic power (Table 3).

IHC for ER and PR has been used for decades now to predict the patient's outcome and response to hormonal therapy (94). Routine clinical practices are generally based on the qualitative status of the IHC expression and categorize samples into negative, weakly positive or positive tumors, although some studies have demonstrated an additional prognostic value of quantitative assessment of ER or PR expression (32, 94–96). In particular, Barllet et al. (95) showed that ER and PR IHC by visual scoring predicted a higher risk of early relapse in hormone receptor-moderate compared to hormone receptor-rich patients (>80–85% for ER, >75% for PR) and demonstrated that patients with a high rate of ER expression might get additional benefit from exemestane. In our study, we found that >74% expression of ER was associated with worse prognosis (*HR* = 3.11, *p* = 0.017) while expression of PR >3% predicted better OS (*HR* = 0.30, *p* = 0.002). Before including the intratumor heterogeneity indicators, we found an independent beneficial prognostic value of PR expression rate in the context of lymph node status (Table 3, Model 1). Similar results were reported by Barllet et al. (95) and Lamy et al. (96), who found the higher PR expression rate was a significant indicator of better prognosis. Nevertheless, a recent overview (94) of 19 studies, which collectively involved 30,754 BC patients, concluded that there is no clear evidence for quantitatively assessed ER and PR as neither prognostic nor predictive marker. Furthermore, they suggested that information on the hormone receptor status beyond “positive” or “negative” should no longer be reported to prevent oncologists subconsciously making different treatment decisions. International studies (97–101) based on the mRNA or IHC data have found that tumors with low expression of ER (1–10%) do not have a significant prognostic impact on survival and benefit from hormonal therapy compared to patients with high ER expression (≥10%). Based on this data, ASCO and CAP recently updated the guideline for ER and PR testing (7) and recommended to report the borderline ER-positive cases (1–10% positive cells) with the additional comment that biologically this type of tumors is more similar to ER-negative cancer and the potential benefit of hormonal therapy is unclear (7). However, a strategy on how to ensure reproducible results of the IHC scoring was not suggested.

Intratumor heterogeneity of PR expression was first measured by Haralick's texture indicators in the present study based on hexagonal grid sampling developed previously for Ki67 studies and subsequently also by AshD bimodality indicator (33–35). This novel data revealed a non-linear relationship between the ratio of PR expression in the tumor tissue and its Haralick's texture entropy (Figure 4). This finding could be explained by the nature of the features extracted (less heterogeneity is observed at the low-minimal and high-diffuse end of the range of expression) and has been reported previously for Ki67 (35). However, the importance of this relationship is highlighted by the finding that PR heterogeneity was an independent predictor of better OS (*HR* = 0.21, *p* = 0.00021) rather than the rate of PR expression *per se*. This is further supported by the prognostic stratification of our patients revealing that lower than 20% and higher than 80% rate of PR expression was associated with worse OS while moderate PR expression (>20% and <80%) was associated with better OS (*p* = 0.0035). While the biological meaning of this non-linearity in the prognostic effect of PR expression remains to be elucidated, it supports the notion that “intratumor heterogeneity is universal, although perhaps non-linear prognostic biomarker” (102). This phenomenon may also explain ambiguous results of the previous efforts to quantify hormone receptor expression for prognostic and predictive modeling with various methodologies and cut-off values.

Bimodality of Ki67 intratumoral distribution, expressed by the AshD indicator, has been reported previously (34) to provide an independent prediction of worse OS and outperforming the rate of Ki67 *per se* in multiple prognostic models. Our current study, performed on a different patient cohort, with IHC slides stained in another laboratory and with the application of slightly different DIA and hexagonal grid analysis settings, provides independent evidence to support this phenomenon. Furthermore, we confirm the independent prognostic role of Ki67 bimodality in the context of PR intratumor heterogeneity, TIL, and clinicopathologic features included in this study of HRBC (*HR* = 3.26, *p* = 0.0062).

Our study does contain some limitations. Firstly, it is based on a relatively small patient cohort with a rather benign course of the disease. Secondly, the retrospective data available about therapy modes did not allow exploring the predictive value of the biomarkers. Nevertheless, we were able to achieve independent prognostic models as a proof-of-concept for computational image-based tissue pathology biomarkers generated from IHC slides. Importantly, the models enabled identification of patients at risk of worse OS in this relatively well-managed disease entity. Of course, large-scale studies with long-term follow-up and therapy data are needed to further validate our findings.

In conclusion, we present a multi-dimensional digital IHC prognostic model for early HRBC, based on three independent cancer pathobiology hallmarks—ssPR expression, proliferation, and immune response. The study revealed that subvisual intratumor heterogeneity indicators of PR and Ki67 expression were more prognostically informative than the rates of their expression. Intratumoral CD8+SATB1+ cell density predicted better OS and could potentially serve as a specific biomarker of anti-tumor immunity. Remarkably, the final prognostic model did not require any other clinicopathologic parameters besides the automatically extracted comprehensive IHC DIA indicators.

## Data Availability Statement

The raw data supporting the conclusions of this article will be made available by the authors, without undue reservation, to any qualified researcher.

## Ethics Statement

The studies involving human participants were reviewed and approved by the Lithuanian Bioethics Committee. The patients/participants provided their written informed consent to participate in this study.

## Author Contributions

DZ, AR, JB, RA, AiL, BP, VO, and ArL participated in the conception and design of the study. DZ and AiL participated in tumor sample collection and IHC staining. DZ and RA performed digital image and statistical analyses. JB carried out the Ki67 digital analysis. DZ in collaboration with AR and ArL, participated in the interpretation of the results and drafted essential parts of the manuscript. All authors critically revised and approved the final version of the manuscript.

## Conflict of Interest

The authors declare that the research was conducted in the absence of any commercial or financial relationships that could be construed as a potential conflict of interest.

## Abbreviations

ASCO: the American Society of Clinical Oncology
AshD: Ashman's D
CAP: the College of American Pathologists
CI: confidence intervals
d: density
DIA: digital image analysis
ER: estrogen receptor
G: histological grade
HER2: human epidermal growth factor receptor 2
HR: hazard ratio
HRBC: hormone receptor-positive breast cancer
IHC: immunohistochemistry
LR: likelihood ratio
OS: overall survival
PD-1: programmed death 1
PD-L1: programmed death-ligand 1
pN: lymph node metastasis status
PR: progesterone receptor
pT: tumor invasion stage
S: stroma compartment
SATB1: special AT-rich sequence-binding protein 1
T: tumor compartment
TIL: tumor-infiltrating lymphocytes
TMA: tissue microarray
TME: tumor microenvironment.
