# Supplementary material for: Independent Prognostic Value of Intratumoral Heterogeneity and Immune Response Features by Automated Digital Immunohistochemistry Analysis in Early Hormone Receptor-Positive Breast Carcinoma
Source: Front Oncol. 2020 Jun 16;10:950. doi: 10.3389/fonc.2020.00950 (PMC7308549; doi:10.3389/fonc.2020.00950)
Supplement: Supplementary file 1 [file Table_1.docx]

Supplementary Material

| **Conventional breast cancer indicators** | | | | | |
| --- | --- | --- | --- | --- | --- |
|  | Mean | Standard deviation | Minimum | Maximum | Median |
| **ER%** | 68.85 | 25.93 | 0.03 | 98.82 | 78.68 |
| **PR%** | 38.50 | 34.11 | 0.03 | 96.28 | 31.87 |
| **HER2%** | 10.53 | 22.20 | 0.002 | 90.62 | 0.64 |
| **Ki67%** | 7.21 | 6.46 | 0.39 | 40.53 | 5.36 |
| **Intratumoral heterogeneity indicators** | | | | | |
| **ER_energy** | 0.24 | 0.28 | 0.02 | 1.00 | 0.14 |
| **ER_homogeneity** | 0.71 | 0.13 | 0.46 | 1.00 | 0.69 |
| **ER_entropy** | 3.48 | 1.53 | 0.00 | 6.00 | 3.67 |
| **ER_contrast** | 1.95 | 1.21 | 0.00 | 5.18 | 1.90 |
| **ER_dissimilarity** | 0.77 | 0.38 | 0.00 | 1.61 | 0.76 |
| **ER_AshD** | 3.36 | 9.24 | 0.82 | 93.12 | 1.86 |
| **PR_energy** | 0.42 | 0.40 | 0.02 | 1.00 | 0.15 |
| **PR_homogeneity** | 0.76 | 0.18 | 0.42 | 1.00 | 0.72 |
| **PR_entropy** | 2.93 | 2.14 | 0.00 | 6.02 | 3.63 |
| **PR_contrast** | 1.76 | 1.69 | 0.00 | 7.25 | 1.68 |
| **PR_dissimilarity** | 0.65 | 0.53 | 0.00 | 1.87 | 0.78 |
| **PR_AshD** | 2.30 | 2.03 | 0.15 | 14.41 | 1.86 |
| **Ki67_energy** | 0.66 | 0.31 | 0.05 | 1.00 | 0.72 |
| **Ki67_homogeneity** | 0.91 | 0.10 | 0.58 | 1.00 | 0.94 |
| **Ki67_entropy** | 1.13 | 1.10 | 0.00 | 4.80 | 0.87 |
| **Ki67_contrast** | 0.24 | 0.36 | 0.00 | 2.29 | 0.13 |
| **Ki67_dissimilarity** | 0.19 | 0.21 | 0.00 | 1.01 | 0.12 |
| **Ki67_AshD** | 2.08 | 1.30 | 0.00 | 7.05 | 1.71 |
| **Immune response indicators** | | | | | |
| **CD8_d_S** | 209.25 | 199.07 | 8.16 | 1243.14 | 152.95 |
| **CD8_d_T** | 70.72 | 85.03 | 0.52 | 451.36 | 37.39 |
| **CD8_SATB1_d_S** | 54.11 | 78.70 | 0.56 | 541.47 | 28.41 |
| **CD8_SATB1_d_T** | 14.99 | 25.87 | 0 | 160.82 | 5.47 |
| **Hypoxia-inducible indicator** | | | | | |
| **HIF1α%_S** | 0.46 | 0.65 | 0.04 | 4.29 | 0.28 |
| **HIF1α%_T** | 0.16 | 0.37 | 0.01 | 3.16 | 0.06 |

Supplementary Table 1: Summary statistics of conventional breast cancer, intratumoral heterogeneity, immune response and hypoxia-inducible indicators: AshD – Ashman’s D, d – density, S – stroma compartment, T – tumor compartment.
