# Supplementary material for: Independent Prognostic Value of Intratumoral Heterogeneity and Immune Response Features by Automated Digital Immunohistochemistry Analysis in Early Hormone Receptor-Positive Breast Carcinoma
Source: Front Oncol. 2020 Jun 16;10:950. doi: 10.3389/fonc.2020.00950 (PMC7308549; doi:10.3389/fonc.2020.00950)
Supplement: Supplementary file 2 [file Table_2.docx]

Supplementary Material

|  | **Factor1** | **Factor2** | **Factor3** | **Factor4** | **Factor5** |
| --- | --- | --- | --- | --- | --- |
| **ER%** | -0.448 | 0.431 | 0.034 | 0.082 | 0.092 |
| **PR%** | -0.161 | 0.835 | -0.056 | -0.011 | 0.233 |
| **Ki67%** | 0.193 | -0.087 | 0.918 | -0.022 | 0.021 |
| **HER2%** | 0.008 | -0.486 | 0.106 | -0.323 | -0.147 |
| **CD8_d_T** | 0.818 | -0.001 | 0.140 | 0.153 | -0.062 |
| **CD8_d_S** | 0.854 | -0.081 | 0.013 | 0.005 | -0.103 |
| **CD8_SATB1_d_S** | 0.814 | 0.036 | 0.103 | -0.145 | -0.091 |
| **CD8_SATB1_d_T** | 0.679 | 0.092 | 0.241 | 0.188 | -0.022 |
| **HIF1a%_S** | 0.444 | -0.137 | 0.212 | -0.128 | 0.391 |
| **HIF1a%_T** | 0.620 | -0.204 | -0.073 | -0.016 | 0.290 |
| **ER_entropy** | 0.051 | 0.173 | 0.007 | 0.848 | -0.155 |
| **ER_AshD** | -0.111 | 0.068 | -0.060 | -0.003 | 0.795 |
| **PR_entropy** | 0.002 | 0.827 | -0.080 | 0.184 | -0.198 |
| **PR_AshD** | 0.048 | 0.626 | -0.065 | -0.273 | -0.207 |
| **Ki67_entropy** | 0.122 | -0.191 | 0.898 | -0.054 | -0.004 |
| **Ki67_AshD** | -0.030 | 0.213 | 0.431 | -0.494 | -0.203 |

Supplementary Table 2: Rotated factor pattern of the conventional breast cancer, intratumoral heterogeneity, immune response and hypoxia-inducible indicators variation: AshD – Ashman’s D, d – density, S – stroma compartment, T – tumor compartment.
