# Supplementary material for: Independent Prognostic Value of Intratumoral Heterogeneity and Immune Response Features by Automated Digital Immunohistochemistry Analysis in Early Hormone Receptor-Positive Breast Carcinoma
Source: Front Oncol. 2020 Jun 16;10:950. doi: 10.3389/fonc.2020.00950 (PMC7308549; doi:10.3389/fonc.2020.00950)
Supplement: Supplementary file 3 [file Data_Sheet_1.docx]

Supplementary Material


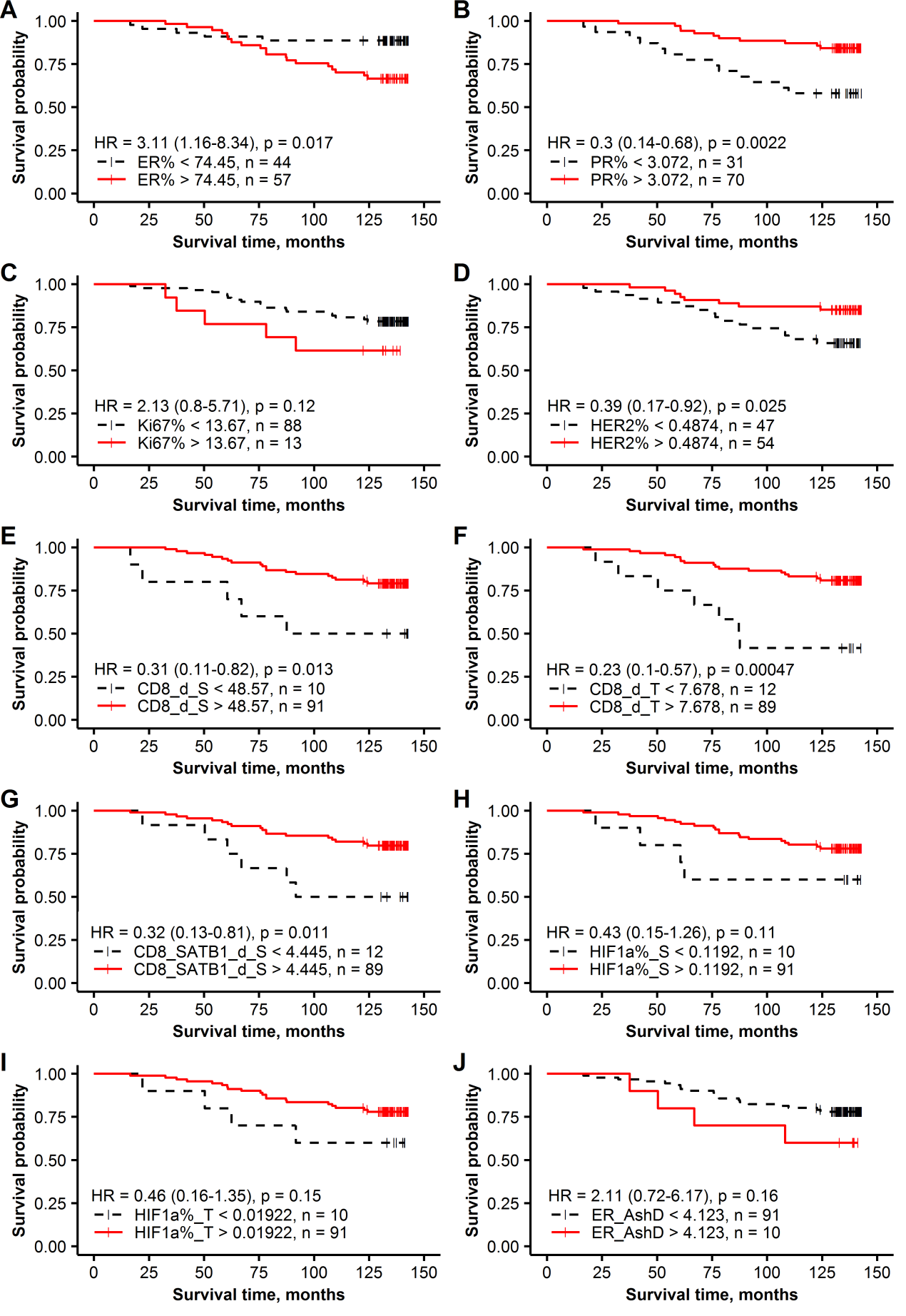


*Continued on the next page*


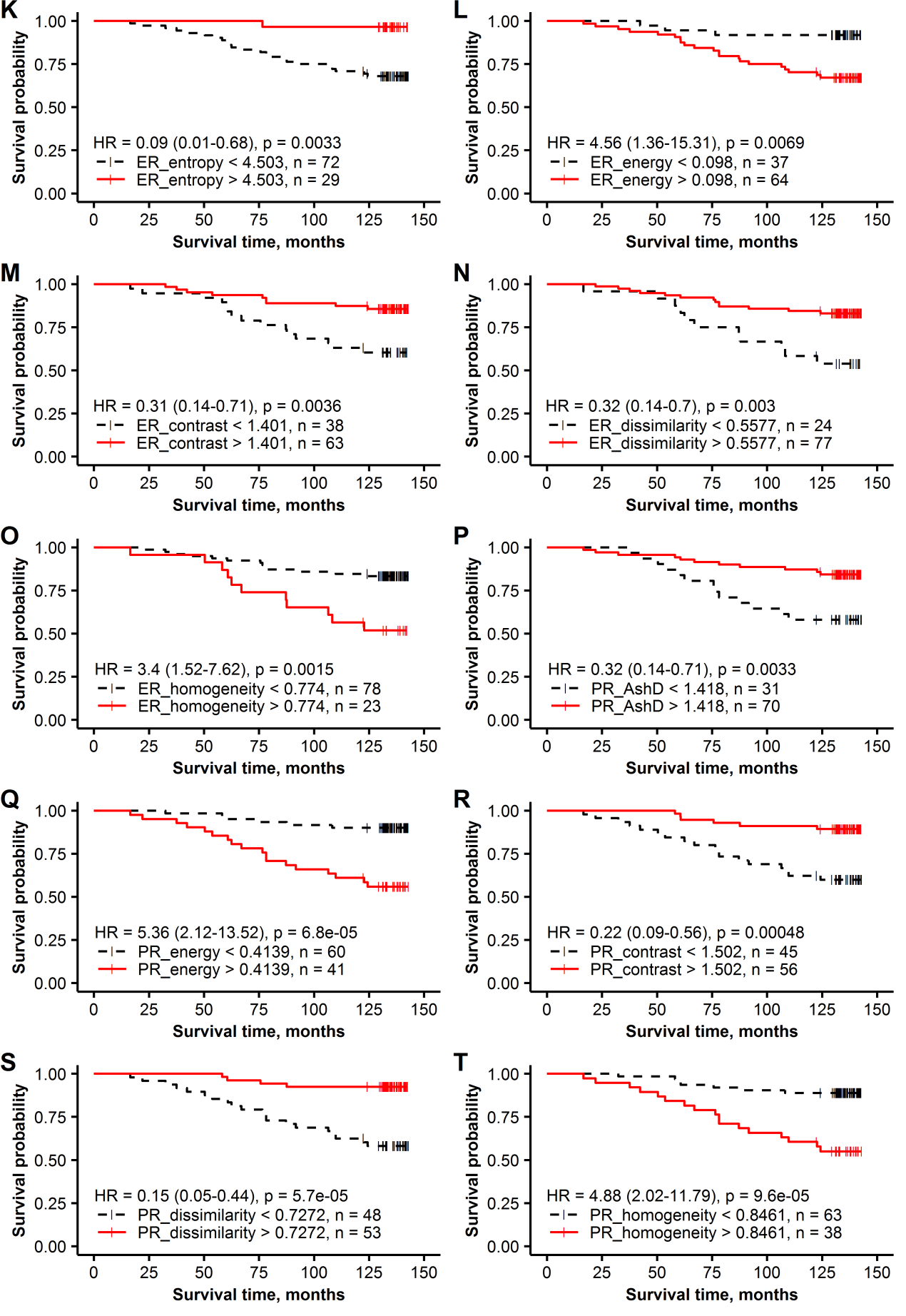


*Continued on the next page*


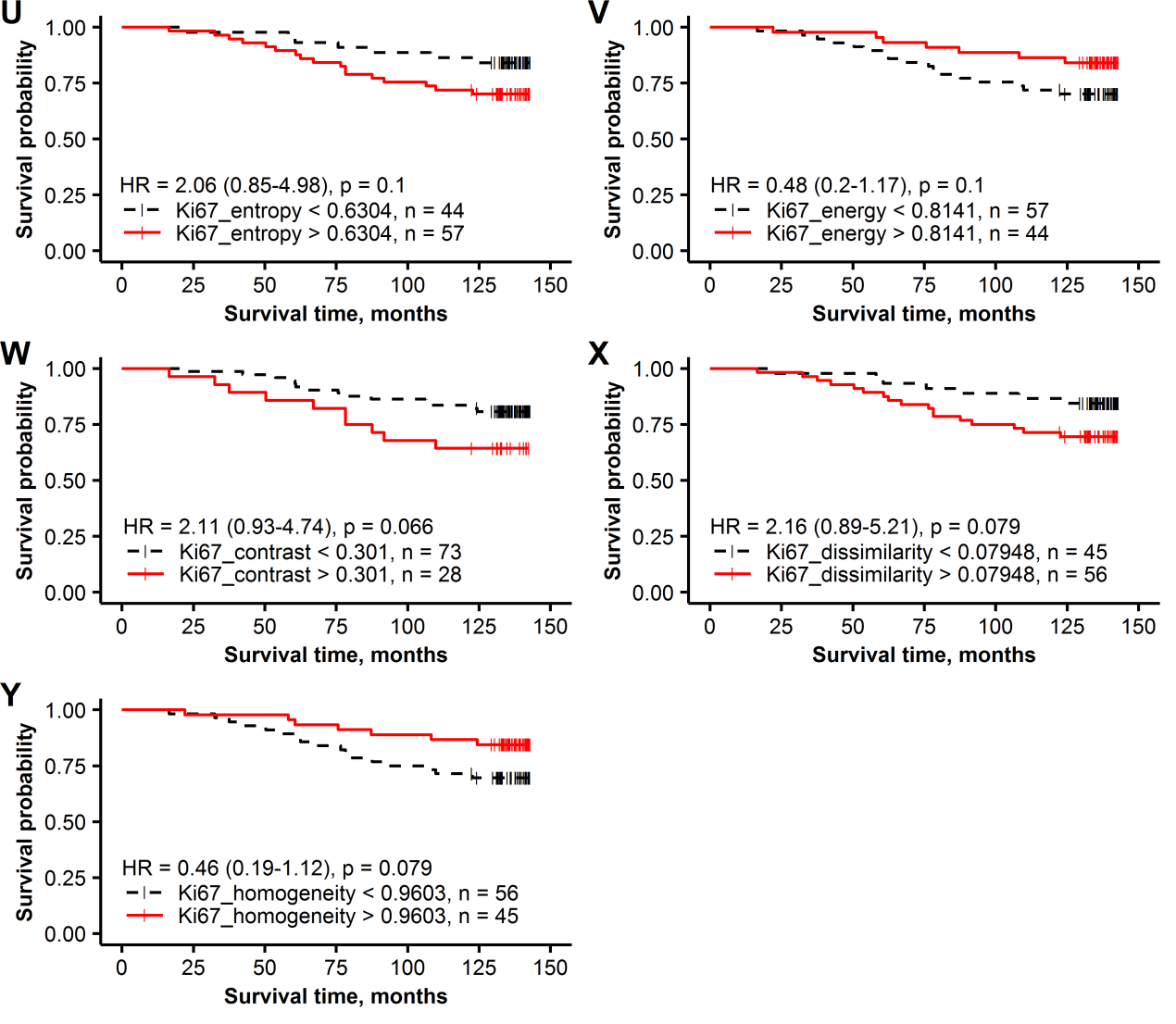


Supplementary Figure 1: Kaplan-Meier survival plots with hazard ratio and log-rank test for correlation of various conventional breast cancer, intratumoral heterogeneity, and immune response indicators with overall survival: AshD – Ashman’s D, d – density, S – stroma compartment, T – tumor compartment.
